# The Trend of Targeted Therapies in Chinese Patients With Ankylosing Spondylitis: Results From a Real-Life Survey

**DOI:** 10.3389/fphar.2021.763707

**Published:** 2021-10-28

**Authors:** Yiquan Wen, Zhuoran Hu, Baozhao Xie, Fei Yuan, Zhengquan Xie, Yutong Jiang, He Lin, Jun Qi, Qiyun Chen

**Affiliations:** ^1^ Division of Rheumatology, The Third Affiliated Hospital of Sun Yat-Sen University, Yuedong Hospital, Meizhou, China; ^2^ Division of Rheumatology, The Third Affiliated Hospital of Sun Yat-Sen University, Guangzhou, China; ^3^ Division of Rheumatology, Wuzhou GongRen Hospital, Wuzhou, China; ^4^ Division of Rheumatology, Dongguan People’s Hospital, Dongguan, China; ^5^ Division of Rheumatology, Panyu Hospital of Chinese Medicine, Guangzhou, China; ^6^ Division of Rheumatology, Fujian Provincial Hospital, Fuzhou, China

**Keywords:** ankylosing spondylitis, biologics, preference, Chinese, cross-sectional study

## Abstract

**Introduction** **:** Targeted medication, including mostly biologics and small-molecule chemical drugs, is an important therapy for ankylosing spondylitis (AS). There are still limited data on the preference of different targeted drugs in Chinese AS patients.

**Methods** **:** A questionnaire-based cross-sectional study was performed on AS patients from six hospitals in three provinces in South China. Anti-rheumatic diseases’ medication history includes the recent and previous usage of biologics or Janus kinase inhibitors (JAKi) in the last complete course of treatment, disease severity, and reasons for targeted-treatment change or preference.

**Results** **:** 354 of 366 participants responded to the online survey. The participants’ median age was 32 years, with a median of 7.3 years of disease duration; 79.7% were male. 63.6% of them were in the course of biologics or JAKi. Generic ETN is the most widely used and willing-to-use biologic though the proportion of its usage shrunk in the present compared with the past. The choice of original-branded ADA demonstrated an increase in usage. The preference of secukinumab and tofacitinib depicted a quick ascending trend.

**Conclusion** **:** TNF-α inhibitors (TNFi) are still the most popular targeted medication for AS in China. Their price influences patients’ preferences mostly. The doctor’s recommendation is also part of the equation. Rheumatologists should pay more attention to patients’ education to formulate targeted therapeutic plans.

## Introduction

Ankylosing spondylitis (AS) is a chronic inflammatory disease, primarily affecting the spine and sacroiliac joints, eventually leading to the loss of spinal mobility (Haroon et al., 2015; Masters et al., 2009). Targeted medication, including mostly biologics and small-molecule chemical drugs, can inhibit the action of specific types of immune-mediated cells or the binding of proteins which play a key role in AS development, including pathways such as tumor necrosis factor (TNF)-α, interleukin (IL)-17A, or Janus kinase (JAK) (van der Heijde et al., 2017).

The anti-TNF treatment has been considered a significant advance in managing patients with AS for many years (Braun et al., 2011). Various sources of antibodies have been applied in routine treatment. The different structures of TNF-α inhibitors (TNFi) have presumably similar mechanisms of action (Ritchlin et al., 2009). There are some kinds of TNFi used in clinical practice worldwide; the most classic components that are also becoming more common currently (Moorkens et al., 2020) are recombinant human TNF receptor–IgG Fc fusion protein, for example, etanercept [ETN (Tracey et al., 2008)], human/mouse chimeric antibodies [infliximab (IFX; Tracey et al., 2008)], and fully humanized antibodies [adalimumab (ADA; Burmester et al., 2013) and golimumab (GLB; Tahir and Kavanaugh, 2018)], which are aimed at the TNFR. The market entry of numerous new generics has brought a significant influence on the usage of biologics because of the reduction in costs, along with interchangeability with the originators in quality, safety, and clinical efficacy (Huang et al., 2017); these might move the preference from the original-branded products toward their generic ones (Glintborg et al., 2018).

Currently, the trend of TNFi in Chinese AS patients is still uncertain, let alone how the emerging secukinumab (Blair 2019) and tofacitinib (van der Heijde et al., 2017) aim to block IL-17A and JAK3 and/or JAK1 (van der Heijde et al., 2017), respectively, and influence the market share of TNFi. There are no reported studies yet on the evolving trends in targeted medication use among AS patients in China. Therefore, the present study would provide some information on patients’ preferences for different targeted drugs from six representative health centers in South China.

## Methods

### Study Design and Study Population

This study was conducted between July and September 2020 in six hospitals from three provinces located in South China, including The Third Affiliated Hospital of Sun Yat-sen University (the principal center); The Third Affiliated Hospital of Sun Yat-sen University, Yuedong Hospital; Dongguan People’s Hospital from Guangdong Province; Fujian Provincial Hospital from Fujian Province; Panyu Hospital of Chinese Medicine; and Wuzhou GongRen Hospital from Guangxi Province. The population of interest aged 18 years or older and with a previous diagnosis of AS, who had been recorded in the database from each center, were invited to answer a 10 min online survey. As no treatment (either active or placebo) was administered to the participants in this study, no ethical committee approval was sought.

### Questionnaire for Patients

Individuals accepting to participate in the online survey were requested to answer an anonymous questionnaire under rheumatologists’ instructions. The questionnaire gathered information on demographic data, time of diagnosis, medication history of anti-rheumatic diseases, disease severity [Bath Ankylosing Spondylitis Disease Activity Index (BASDAI; Garrett et al., 1994)], and reasons for treatment change (see the Supplementary Material).

### Objectives

The objectives of the present study were targeted medication history of patients and their preference for and reasons for the usage of the above-mentioned drugs. The relevant medication history includes the recent and previous usage of biologics or JAKi in the last complete course of treatment.

### Statistical Analysis

The presentation of the data is descriptive. Continuous data are presented as mean ± standard deviation (SD) or median with interquartile range (IQR) as appropriate. Categorical variables are presented as frequency counts with percentages. R (version 3.6.1) was utilized, and statistical significance was assumed at the *p* < 0.05 level. Power analysis will be performed with the “pwr” package. The significance level (1-α) and power (*β*) will be set as 0.05 and 0.9, respectively.

## Results

### Baseline Characteristics

The questionnaire recorded the data of 366 patients with AS. The response rate was 96.7% (354/366). The baseline data are shown in Table 1. The participants’ median age was 32 years, with a median of 7.3 years of disease duration; 79.7% were male. 60.2% of patients were under inactive conditions (BASDAI<4). 63.6% of them were in the course of biologic or JAKi treatment. 39 (11%) reported that they had never used biologics. After power analysis with the effect size (0.298), the PASS software calculated that a total sample size of at least 143 would suffice. Non-steroidal anti-inflammatory drugs (NSAIDs) were the most common non-biologic medication (54.5%). Missing data did not occur since questions in our questionnaire were compulsory.

**TABLE 1:** Characteristics of the participants.

Characteristics	*N* = 354
Age, median (IQR) (years)	32.0 (27.0; 38.0)
Gender, yes, n (%)	
Male	282 (79.7)
Income	
No salary	125 (35.3)
≤5,000	140 (39.6)
5,000–10,000	58 (16.4)
10,000–20,000	23 (6.5)
≥20,000	8 (2.3)
Disease duration, median (IQR) (years)	7.30 (2.76; 12.51)
Diagnostic delay, median (IQR) (years)	1.09 (0.30; 4.08)
BASDAI, median (IQR)	3.3 (2.3; 4.9)
State of the disease, n (%)	
Inactive, BASDAI < 4	213 (60.2)
Active, BASDAI ≥ 4	141 (39.8)
Extraarticular symptoms, n (%)	
Uveitis	52 (14.7)
Inflammatory bowel disease	54 (15.3)
Sausage-like fingers or toe	8 (2.3)
Enthesitis	86 (24.3)
Biologics’ history, n (%)	
Never	39 (11.0)
Withdrawn or changed	90 (25.4)
Being under treatment	225 (63.6)
Recent non-biologic medication, n (%)	
None of any medication	15 (4.2)
NSAIDs	193 (54.5)
cDMARDs	72 (20.3)
Traditional Chinese medication	24 (6.8)

### The Adherence of Biologics’ Usage

**FIGURE 1:**
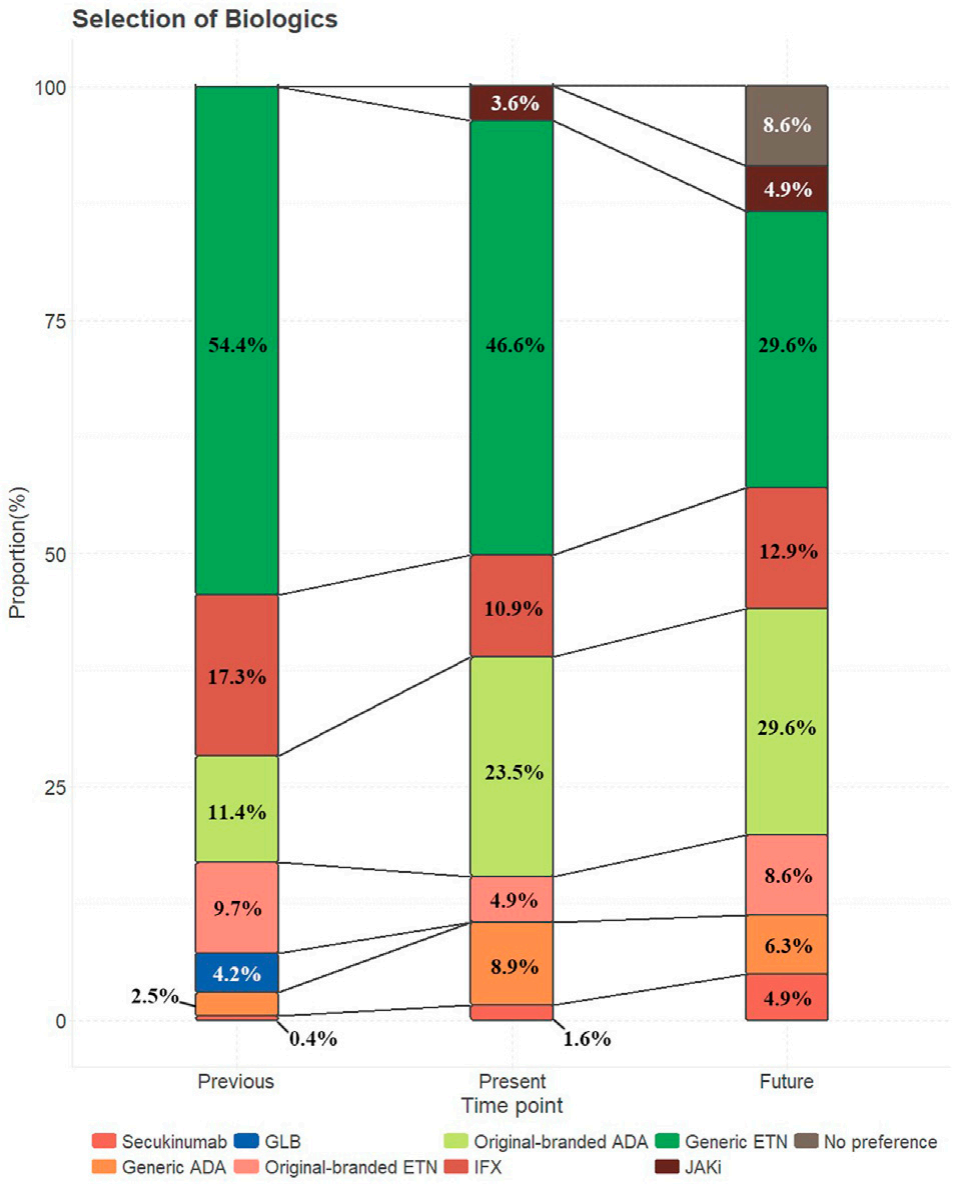
Choice or preference of biologics in different time points.

As shown in Figure 1, after excluding patients who never had biologics, generic ETN is the most used and willing-to-use biologic though the proportion of its usage shrunk, from 54.4% of participants in the past to 46.6% in the present and then to 29.6% still preferring to choose it if AS recurred in the future. 8.6% of participants declared that they did not have a specific preference for biologics.

Original-branded ADA demonstrated more than a double increase in the presence and growth stably when it comes to the future. The preference of secukinumab (Blair, 2019) and tofacitinib depicted a similar, quick ascending trend.

Contrary to generic ADA, which had an inverted “U-shape” trend, ETN and IFX showed a “U-shape” one. GLB was used in 10 (4.2%) of participants previously, but none of them continue to use it currently or would choose GLB in the future.

### The Reasons for Biologics’ Change/Withdrawal and Preference

**FIGURE 2:**
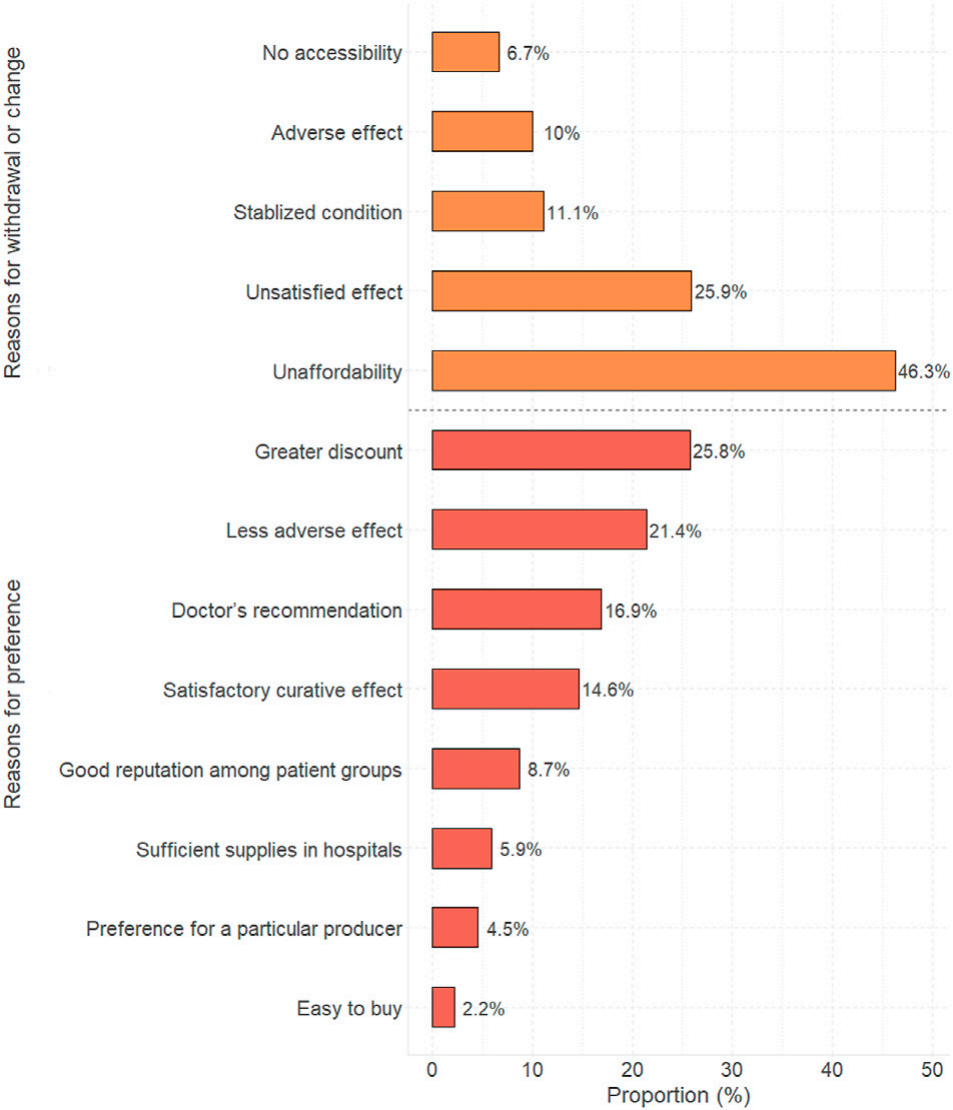
Proportion of reasons for withdrawal/change of or preference for biologics.

After excluding those who had never used biologics, as shown in Figure 2, the most mentioned reason for withdrawal or change is “unaffordability” (46.3%). Unsatisfied effect (25.9%) or adverse effect (10%) was counted as the third of all reasons. “Not accessible to the biologics in the primary health center” was chosen by 6.7% of participants who underwent biologics’ change/withdrawal. It was shown that some novel biologics are difficult to get in the primary health center so that these patients have to choose another one.

Followed by “less adverse effect” (21.4%), more than a quarter of participants (25.8%) recognized that greater discount (namely, being more affordable) is an important, influential factor when choosing biologics. The doctor’s recommendation (16.9%) and “satisfactory curative effect” (14.6%) are also frequently mentioned reasons for preference. The reasons for withdrawal of or preference for these drugs are given in Supplementary Figures S1, S2.

## Discussion

This study found that most patients with AS in South China preferred to use TNFi over time, whether they were domestically produced generic or original-branded and even if other novel biologics partially occupied their dominance. Among all sorts of TNFi, ETN, IFX, and their domestically generic drugs were still the first choice for most AS patients.

Original-branded ADA demonstrated more than a double increase in the presence and growth stably when it comes to future preference. It has been becoming popular since it was first marketed in China for AS in 2010; however, although domestically generic ADA witnessed a 3.6-fold increase after its marketing in the home in 2019, the patients’ present preference for it was still tepid.

Secukinumab (Blair, 2019) and tofacitinib showed a preemptive trend in patients’ preference. Secukinumab, which demonstrated a favorable safety profile over long-term treatment in patients with AS and psoriatic arthritis (PsA) (Baraliakos et al., 2020; Deodhar et al., 2019), was approved by the Chinese National Medical Products Administration (NMPA) for treating refractory AS in April 2020 (https://www.novartis.com.cn/news/nuo-hua-ke-shan-ting-si-ku-qi-you-dan-kang-qiang-zhi-xing-ji-zhu-yan-gua-ying-zheng-huo-pi, in Chinese). This biologic has been noted in some participants in our cohort in a short time. Tofacitinib entered the Chinese market in 2017 (http://list.cde.org.cn/index/detail/id/522, in Chinese) as a medication for rheumatoid arthritis (RA) (Blair, 2019). Although there has been a phase II clinical trial that revealed tofacitinib has a satisfactory clinical efficacy for AS (van der Heijde et al., 2017), AS is still not an indication of tofacitinib in China. However, in our study, nine participants reported that they have used tofacitinib in “the present.”

Affordability was the primary associated factor for patients’ preference. That is the reason why generic ETN is in our study since it was included in the Chinese National Reimbursement Drug List (part B) in 2017, but ETN was not until March 2021 (http://bmfw.www.gov.cn/ybypmlcx/index.html). Contrarily, though ETN and IFX have reduced prices due to the fierce market competition, they are still much more expensive than domestic generics. Therefore, an evident decrease in the presence and ascending preference were observed for original-branded ETN. These U-shape trends might be partly owing to the ongoing, nationwide inclusion of health coverage after 2020. Due to a substantial discount before entering the above-mentioned list in 2020, the preference for original-branded ADA keeps increasing, but not for generic ADA. The unsatisfactory effect could count on the decline (see Supplementary Figure S1).

Notably, except for prices and efficiency, the doctor’s recommendation and reputation among patients also impact patients’ choice, which reflects the inadequate literacy of patients using the targeted medication. Accessibility is becoming less important, presumably due to the rapid development of the domestic logistics industry (http://www.chinawuliu.com.cn/lhhzq/202102/23/541764.shtml, in Chinese).

Overall, our study showed that TNFi are still the most popular targeted medication for AS in China, especially the domestically produced, generic drugs of ETN. The price influences patients’ preference mostly, followed by curative efficiency and adverse effects. With more generic biologics rushing in the market and with the original-branded drugs lowering their prices or entering the health coverage, patients would have more active choices. The doctor’s recommendation is also part of the equation. Therefore, in clinical practice, rheumatologists should pay more attention to patients’ education to formulate targeted therapeutic plans.

There are several limitations of our study. First, we cannot exclude the possibility of patient selection bias because centers that participated in this study were tertiary referrals in South China; thus, results from this study cannot represent the actual situation in China. More large, nationwide surveys assessing the AS patient’s preference for targeted medication and reasons may provide us a new perspective for patient-oriented treatment.

### Key Messages

#### What Is Already Known About This Subject?

Targeted medications are an important therapy for ankylosing spondylitis. There are numerous choices of these drugs.

#### What Does This Study Add?

In this study, we provided some information on the evolving trend of some representative biologics from the perspective of Chinese patients. Moreover, we dug in the reasons for and influential factors of patients’ preference for targeted medications.

#### How Might This Impact on Clinical Practice or Future Developments?

The price influences patients’ preference mostly. Also, the doctor’s recommendation is also an impact factor. Therefore, in clinical practice, rheumatologists should pay more attention to patients’ education to formulate targeted therapeutic plans.

## Data Availability

The raw data supporting the conclusions of this article will be made available by the authors, without undue reservation.
