# Supplementary material for: The Trend of Targeted Therapies in Chinese Patients With Ankylosing Spondylitis: Results From a Real-Life Survey
Source: Front Pharmacol. 2021 Oct 28;12:763707. doi: 10.3389/fphar.2021.763707 (PMC8581396; doi:10.3389/fphar.2021.763707)
Supplement: Supplementary file 1 [file DataSheet1.docx]

**SUPPLEMENTARY MATERIALS**

**questionnaire information**

1. Name

2. Gender

3. Age (years old)


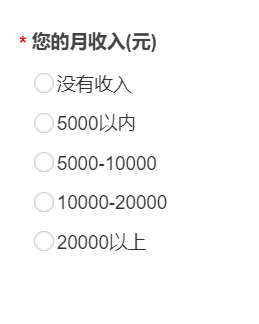
4. Income (Categorial)

‘5000~10000 Yuan’

‘10000~20000 Yuan’

‘>20000 Yuan’

‘No income’

‘<5000 Yuan’

5. When were you diagnosed with ankylosing spondylitis？ (yyyy /mm)

6. When have you had low back pain or other symptoms of AS? (years, with months, is preferable if appropriate) ______years______months

7. Have you been having symptoms below within the last 10 days? ‘1’ is no symptoms, ‘10’ is quite severe.


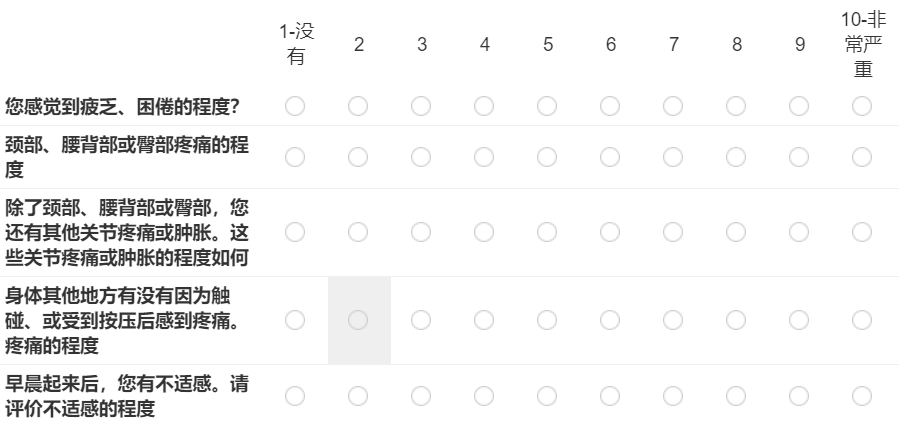


1: None.

10: Quite severe.

The degree of fatigue

c

The degree of pain and/or swollen in other joints excepts neck, back and/or hip.

The degree of malaise after getting up in the morning.

The degree of pain in neck, back and/or hip.


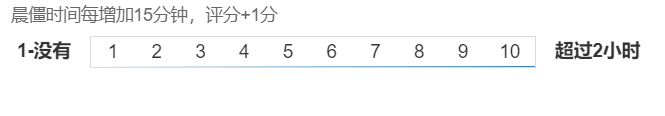
8. 晨起腰背部或其它关节僵硬持续时间 The duration of joints or back stiffness after getting up in the morning. Each additional 15 minutes is worth 1 score.

10: Over 2 hours.

1: None.

9. Have you ever been diagnosed with the below extraarticular manifestations? (multiple choice)


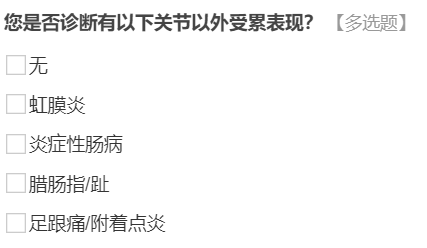


NSAIDs

e.g., voltaren, celecoxib, diclofenac, meloxicam, etc.

Sausage-like figures and/or toes.

Inflammations in attachment points.

Inflammatory bowel disease.

Iritis.

None.


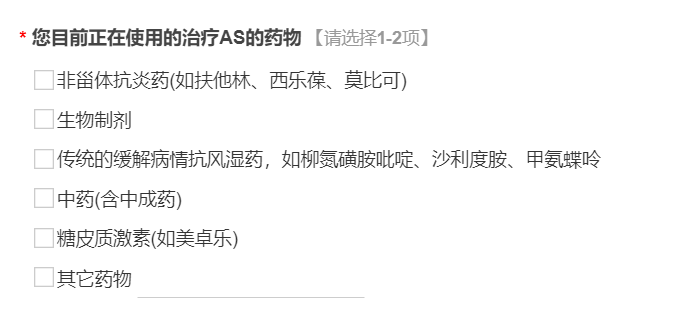
10. The present medication for AS. (multiple choice)

Other medication. (fill)

Glucocorticoid, e.g., methylprednisolone.

Traditional Chinese medicine.

Conventional DMARDs,

e.g.,sulfasalazine, thalidomide, methotrexate, etc.

Biologics.

11. Which biologics are you using below in the present? (Single choice)


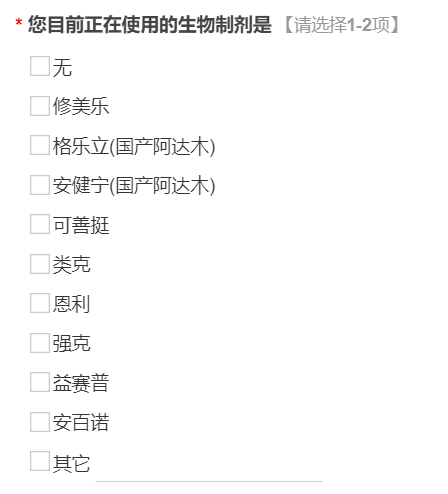


Other biologics.

TNFi (domestic) and its brand name.

TNFi (domestic) and its brand name.

TNFi (domestic) and its brand name.

Etanercept (imported) and its brand name.

Infliximab (imported) and its brand name.

Secukinumab and its brand name.

Adalimumab (domestic) and its brand name.

Adalimumab (domestic) and its brand name.

Adalimumab (imported) and its brand name.

No biologics.

12. Which biologics below did you ever withdraw? (multiple choice)


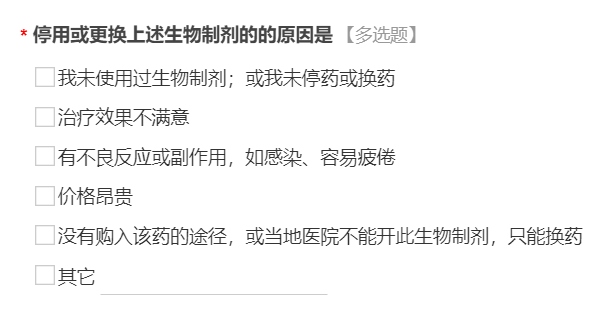

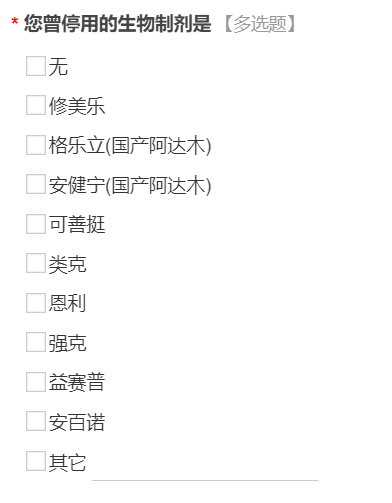
13. The reasons you withdrew or changed the biologics chose in the last question. (multiple choice)

Not accessible in primary health centers.

Other biologics. (fill)

Other reasons. (fill)

Unaffordable.

Adverse effects or side effects,

e.g., infection and fatigue.

Unsatisfactory effects.

I did not change or withdraw biologics;

or I did not ever use biologics.

TNFi (domestic) and its brand name.

TNFi (domestic) and its brand name.

TNFi (domestic) and its brand name.

Etanercept (imported) and its brand name.

Infliximab (imported) and its brand name.

Secukinumab and its brand name.

Adalimumab (domestic) and its brand name.

Adalimumab (domestic) and its brand name.

Adalimumab (imported) and its brand name.

No biologics.

14. Which biologics do you prefer (without regard for price)? (multiple choice)

15. The influential factors for your preference. (multiple choice)


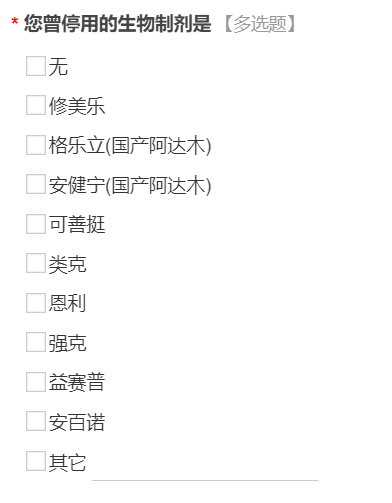

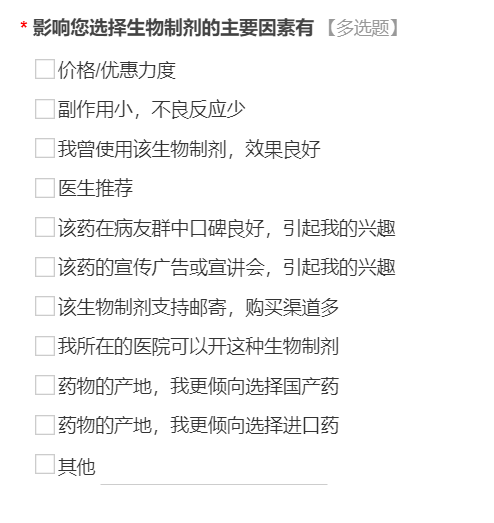


Other reasons. (fill)

Preference for imported medication.

Preference for domestic medication.

Easy to get from the primary health center.

Easy to get by express.

Attractive advertisements.

Good reputation.

Recommendation from specialists.

Satisfactory effects.

Less adverse effects or side effects.

Price / discount.

Other biologics. (fill)

TNFi (domestic) and its brand name.

TNFi (domestic) and its brand name.

TNFi (domestic) and its brand name.

Etanercept (imported) and its brand name.

Infliximab (imported) and its brand name.

Secukinumab and its brand name.

Adalimumab (domestic) and its brand name.

Adalimumab (domestic) and its brand name.

Adalimumab (imported) and its brand name.

No preference.

**
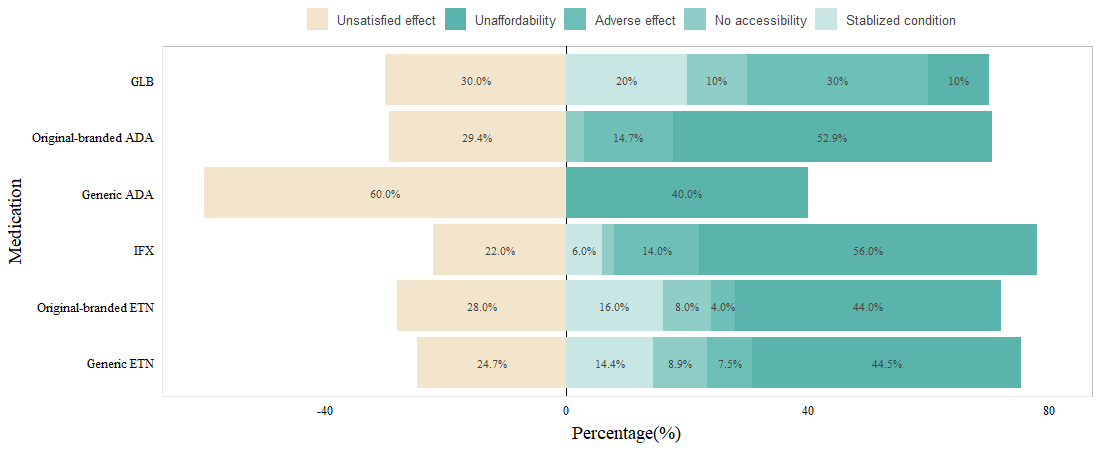
Supplementary figures**

Supplementary Figure 1 The reason of biologics withdrawal.


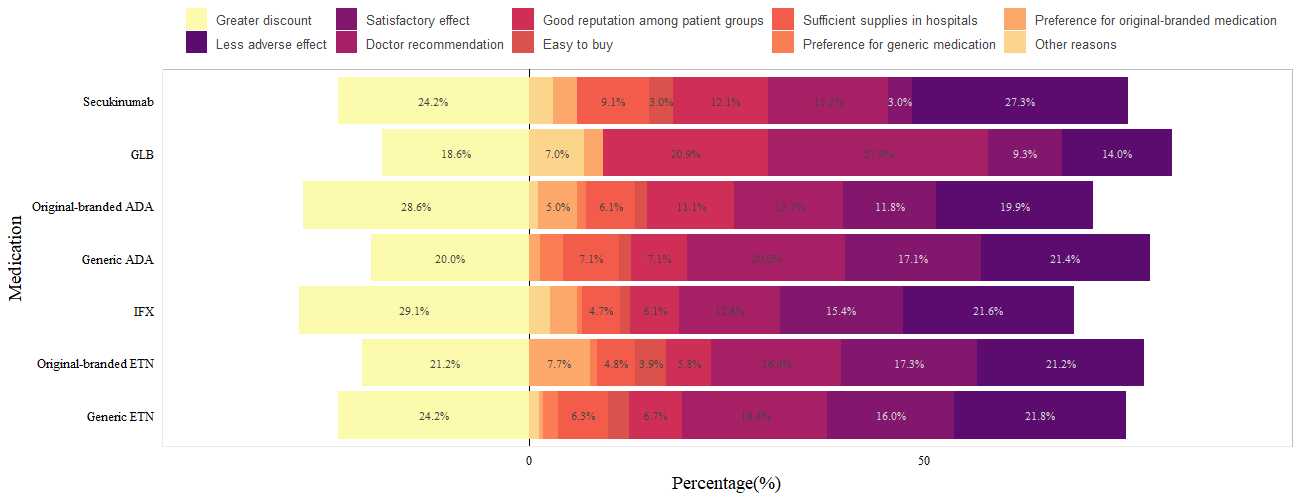


Supplementary Figure 2 The reason of biologics preference.


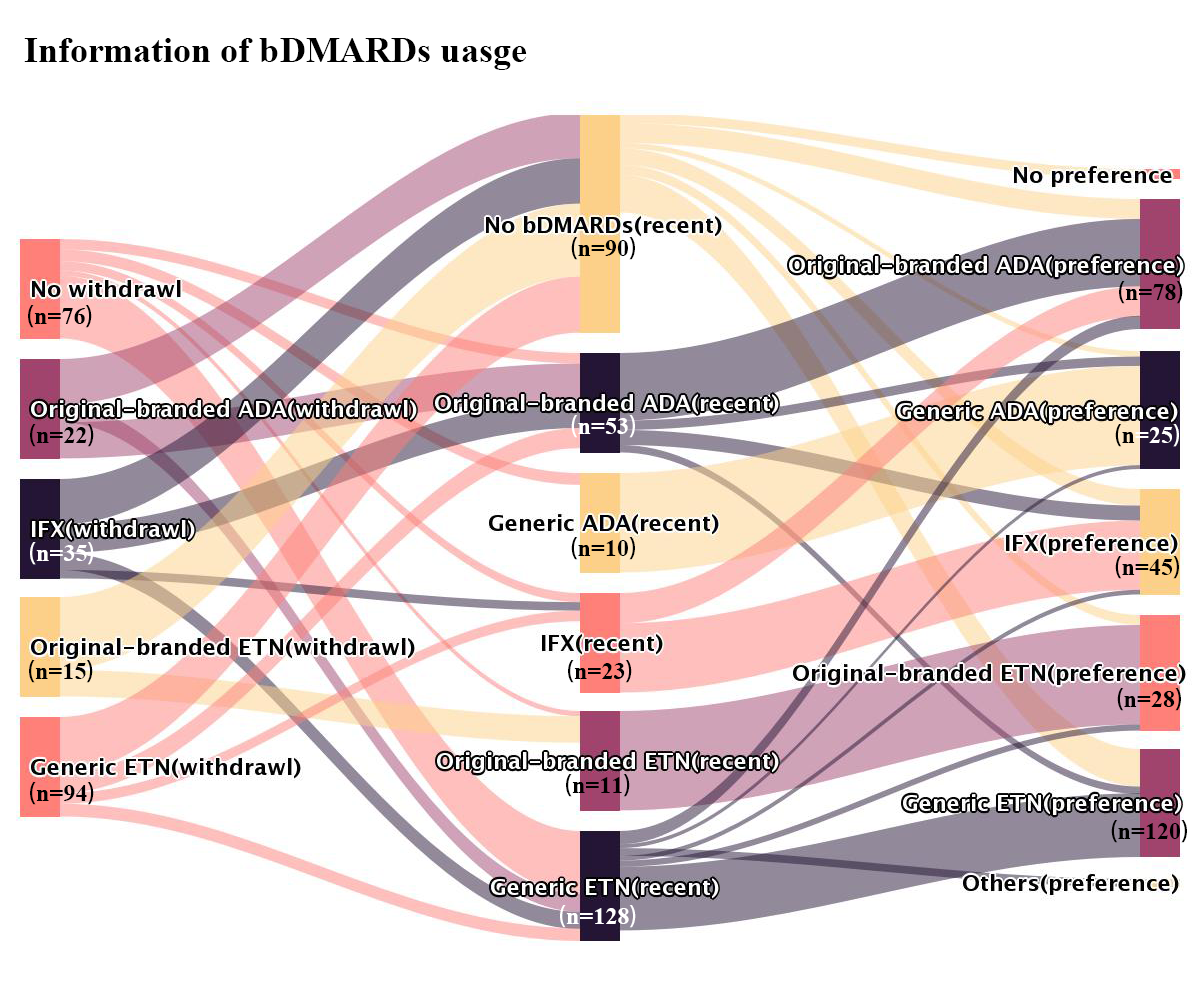


Supplementary Figure 3 The flow of using biologics history and preference.
